# Formulary

**Published:** 1880-02

**Authors:** Don Roy


FORMULARY.—*Gonorrhœa.*—The following is the best thing I have ever used for gonorrhœa:

℞.—Cubebs, pulverized..............6 drachms
Soda bicarb.....................5 drachms
Make twelve powders.

Mix. One powder in water three times a day.

*Don Roy, M. D.*

---

The following is a reliable remedy for spermatorrhœa:

℞.—Aconitin.........................1 grain
Lupulin..........................48 grains

Mix. Divide in sixteen powders. Dose, one at bedtime.

The aconitin above should not be confounded with the alkaloid aconita, which is too powerful.—*Southern Clinic.*

